# A novel efficient strategy to generate liver sinusoidal endothelial cells from human pluripotent stem cells

**DOI:** 10.1038/s41598-024-64195-1

**Published:** 2024-06-15

**Authors:** Shang-Ping Tian, Jian-Yun Ge, Yu-Mu Song, Xiao-Qing Yu, Wen-Hao Chen, Yu-Ying Chen, Di Ye, Yun-Wen Zheng

**Affiliations:** 1 Guangdong Provincial Key Laboratory of Large Animal Models for Biomedicine, and South China Institute of Large Animal Models for Biomedicine, School of Pharmacy and Food Engineering, Wuyi University, Jiangmen, Guangdong China; 2 State Key Laboratory of Bioreactor Engineering, East China University of Science and Technology, Shanghai, China; 3 Institute of Regenerative Medicine, and Department of Dermatology, Affiliated Hospital of Jiangsu University, Jiangsu University, Zhenjiang, Jiangsu China; 4 Department of Medical and Life Sciences, Faculty of Pharmaceutical Sciences, Tokyo University of Science, Noda, Chiba Japan; 5 Institute of Medical Science, Center for Stem Cell Biology and Regenerative Medicine, The University of Tokyo, Tokyo, Japan

**Keywords:** Cell biology, Developmental biology, Stem cells

## Abstract

Liver sinusoidal endothelial cells (LSECs) are highly specialized endothelial cells (ECs) that play an important role in liver development and regeneration. Additionally, it is involved in various pathological processes, including steatosis, inflammation, fibrosis and hepatocellular carcinoma. However, the rapid dedifferentiation of LSECs after culture greatly limits their use in vitro modeling for biomedical applications. In this study, we developed a highly efficient protocol to induce LSEC-like cells from human induced pluripotent stem cells (hiPSCs) in only 8 days. Using single-cell transcriptomic analysis, we identified several novel LSEC-specific markers, such as *EPAS1, LIFR, and NID1*, as well as several previously revealed markers, such as *CLEC4M, CLEC1B, CRHBP* and *FCN3*. These LSEC markers are specifically expressed in our LSEC-like cells. Furthermore, hiPSC-derived cells expressed LSEC-specific proteins and exhibited LSEC-related functions, such as the uptake of acetylated low density lipoprotein (ac-LDL) and immune complex endocytosis. Overall, this study confirmed that our novel protocol allowed hiPSCs to rapidly acquire an LSEC-like phenotype and function in vitro. The ability to generate LSECs efficiently and rapidly may help to more precisely mimic liver development and disease progression in a liver-specific multicellular microenvironment, offering new insights into the development of novel therapeutic strategies.

## Introduction

The liver plays a crucial role in maintaining homeostasis, with hepatic homeostasis being regulated by the dynamic interplay between hepatocytes and nonparenchymal cells (NPCs). The cellular interactions have been proven to be essential for supporting the overall function of the liver^1^. The NPCs include liver sinusoidal endothelial cells (LSECs), hepatic stellate cells (HSCs), cholangiocytes, and Kupffer cells. To date, LSECs have been the least studied cell type in the liver, but a growing number of recent studies have identified their key role in liver development, homeostasis and pathophysiology^2^. As a unique endothelial cell type in the liver, LSECs link the portal and central veins in the hepatic sinusoids and provide nutrients to liver cells as well as transcellular fenestrations arranged in sieve plates, which enables the selective transport of nutrients and scavenger functions^3^. LSECs have also been shown to play an important role in hepatic regeneration during liver injury by regulating HSCs quiescence and providing signals that initiate and maintain Kupffer cells^4–6^. LSECs not only play a crucial role in the development, homeostasis, and pathophysiology of the liver but also have been found to be associated with various liver diseases. Functional abnormalities in LSECs disrupt hepatic hemodynamics in diseases such as cirrhosis and liver fibrosis, affecting the normal structure and function of the liver^7^. Furthermore, alterations in LSEC function have been implicated in the formation and progression of malignant tumors, including liver cancer. However, until recently, the origin and developmental trajectory of LSECs during liver development and regeneration have remained unclear. Moreover, the specific markers that can be used to identify and purify LSECs are still controversial^8–10^. Conventional in vitro culture methods often make it difficult to obtain large quantities of purified LSECs, while the loss of cellular function also limits their application^11,12^. Hence, there is an urgent need to develop a rapid and efficient LSEC induction and culture strategy.

Human induced pluripotent stem cells (hiPSCs) are similar to embryonic stem cells (ESCs) in terms of morphology, gene and protein expression, and multilineage differentiation capacity. Importantly, hiPSCs provide a reliable source for biomedical applications. To date, several groups shave partially succeeded in generating LSECs from pluripotent stem cells (PSCs) in both mice and humans. Arai et al.^13^ showed that adrenomedullin and transforming growth factor b (TGF-b) inhibitors led to increased expression of LSEC-related genes in the mouse embryoid body (EB). Koui et al.^14^ successfully induced human liver sinusoidal endothelial-like cells by inhibiting TGF-β signaling in hiPSCs. Gage et al.^15^ demonstrated that venous progenitors respond more rapidly and robustly than arterial cells to upregulate LSEC lineage markers in response to cyclic AMP (cAMP), TGF-β inhibition and hypoxia. However, the LSEC-like cells generated in these studies required complicated procedures, had a long induction cycle and were difficult to reproduce, thus limiting their practical application.

In this study, using a novel two-step strategy, we first induced hiPSCs to differentiate into endothelial progenitor cells (EPCs) and further into LSEC-like cells in just 8 days at approximately 80% purity. We found that KDR^+^ CD34^+^ EPCs may be crucial precursors for LSECs. In conclusion, our protocol provides an efficient and convenient way to generate hiPSC-derived LSEC-like cells, which may facilitate future applications in liver regeneration and metabolic studies, disease modeling and drug testing.

## Results

### Efficient differentiation of hiPSCs into induced endothelial progenitor cells (iEPCs)

**Figure 1:**
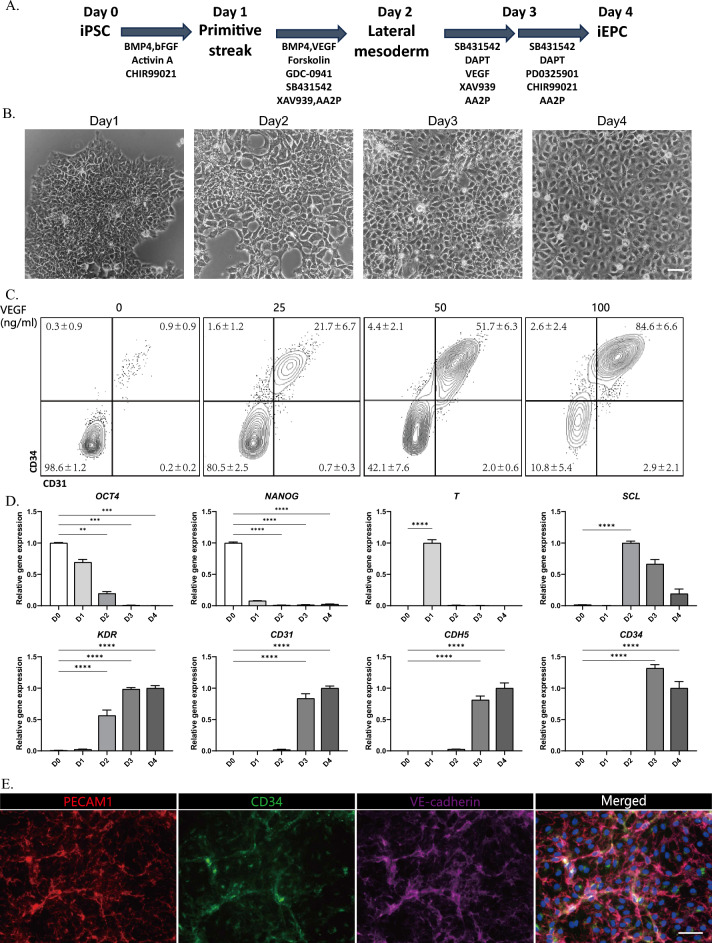
Rapid process to differentiate iPSCs into iEPCs. (**A**) Schematic representation of the protocol for the differentiation of hiPSCs to iEPCs. (**B**) The cell morphology from day 1 to day 4 during differentiation. Scale bar, 50 μm. (**C**) The cells were treated with different concentrations of VEGF (0 ng/ml, 25 ng/ml, 50 ng/ml, or 100 ng/ml) from days 2–3. Representative FACS plots for iPSC-derived cells on day 4 are shown. (**D**) Changes in the gene expression of *OCT4, NANOG, T, SCL, KDR, PECAM1, CD34* and *CDH5* during differentiation. The results are shown as the mean ± SD of 3 independent experiments. (**E**) Immunofluorescence staining for CD31, CD34, and VE-CADHERIN on day 4. Nuclei were counterstained with DAPI. Scale bar, 50 μm. *** p < 0.001 and **** p < 0.0001.

It is well known that EPCs arise from lateral mesodermal cells during embryogenesis. We developed an efficient system for differentiating EPCs based on published protocols with some modifications^16,17^. Figure 1A illustrates the 4-day iEPC differentiation protocol. Shortly after 4 days of induction, the proportion of endothelial-like cells increased significantly (Fig. 1B), as evidenced by a cobblestone-like morphology with flattened cell bodies. Moreover, the concentration of VEGF significantly influenced the production of endothelial cells (ECs). Flow cytometry analysis revealed that the absence of VEGF led to minimal production of CD31^+^CD34^+^ ECs, and a low concentration of VEGF (25 ng/ml) yielded less than 25% CD31^+^CD34^+^ ECs. In contrast, a high concentration of VEGF (100 ng/ml) resulted in a proportion exceeding 80% (Fig. 1C). Therefore, to enhance the efficiency of generating endothelial-like cells, we opted to use a high concentration of VEGF (100 ng/ml) in subsequent experiments. We monitored gene expression dynamics during differentiation using qRT‒PCR and observed a gradual decrease in *OCT-4* and *NANOG* after day 1. The mesodermal markers T and SCL gradually decreased after days 1–2. In contrast, the levels of the mesodermal and EPC marker *KDR* increased beginning on day 2 and peaked on day 4. The endothelial cell markers *CD31*, *CD34* and *CDH5* appeared from the day 3 and reached higher levels on day 4 (Fig. 1D). Moreover, the protein expression of the endothelial cell (EC) marker platelet endothelial adhesion molecule 1 (PECAM1), hematopoietic progenitor cell antigen CD34, and vascular endothelial cell adherent junction protein (VE-cadherin) in iEPCs was confirmed by fluorescent immunostaining (Fig. 1E).

Taken together, we succeeded in inducing EPCs with a purity as high as 80% in 4 days using a modified method from a previous report involving the combination of growth factors and chemical small molecules. The iEPCs shared similar markers with LSEC precursor markers as previously reported and were used as potential intermediates in this study for subsequent LSEC induction^14^.

### Generation of hiPSC-derived LSECs

**Figure 2:**
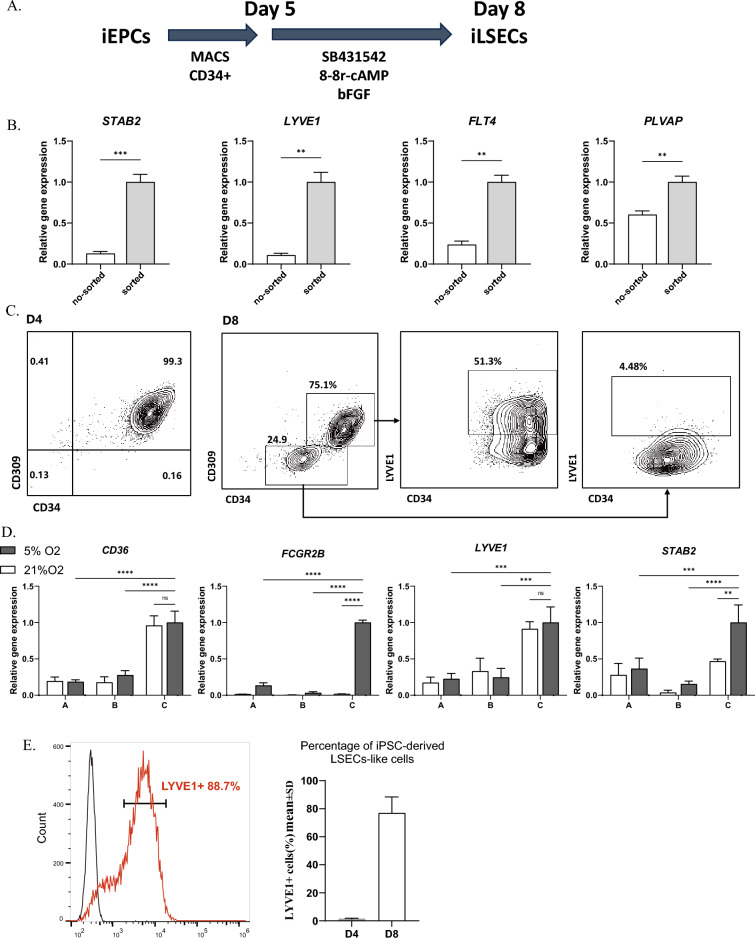
Generation of iLSECs from iEPCs. (**A**) Schematic of the differentiation from hiPSC-derived EPCs into LSECs. (**B**) Expression levels of LSEC gene markers in CD34 sorted and no-sorted cells at D8. The results are shown as the mean ± SD of 3 independent experiments. **p < 0.01, ***p < 0.001. (**C**) FACS analysis of differentiated LSECs. Representative plots are shown. (**D**) Gene expression of *STAB2, LYVE1, FCGR2B,* and *CD36* in hiPSC-derived LSEC-like cells cultured in differentiation medium A, B and C under hypoxic (5% O2) or normoxic conditions (21% O2). The results are shown as the mean ± SD of 3 independent experiments. ** p < 0.01, *** p < 0.001, **** p < 0.0001 and ns not significant. (**E**) Percentages of LYVE1^+^ cells at D4 and D8 (n = 5). The results are shown as representative plots for purified D8 cells. On D4, 0.92 ± 0.57% of the cells were LYVE1^+^ cells, and on D8, 76.94 ± 5.16% were LYVE1 + cells.

To generate LSEC-like cells, we treated iEPCs with pathway modulators involved in LSEC specification, including 8-Br-cAMP (cAMP), a known mediator of adrenomedullin signaling; the TGF-β inhibitor SB431542; and fibroblast growth factor 2 (bFGF) (Fig. 2A)^18,19^. To further enrich the purity of the LSEC-like cell population, we isolated differentiated CD34 positive cells by magnetic-activated cell sorting (MACS) on day 4 and examined their gene expression patterns following LSEC specification. After 4 days of maturation, compared to no-sorted population, the CD34^+^ population had a greater proportion of CD31 and CD309 double positive cells and exhibited much greater expression of LSEC-specific genes such as *STAB2, LYVE1, FLT4* and *PLVAP* (Fig. 2B). To promote the differentiation of the purified iEPCs to LSECs, approximately 20% of the day 4 CD34^+^KDR^+^ iEPC population lost KDR expression by day 8. The remaining KDR positive iEPCs could be further differentiated to generate approximately 50% LYVE-1^+^ LSEC-like cells; in contrast, the KDR negative iEPCs hardly generated LYVE-1^+^ cells. Thus, we suggest that KDR may serve as a crucial marker for LSEC specification. (Fig. 2C). To further enhance the generation of LSEC-like cells, we compared the effects of three differentiation medium under hypoxic (5% O2) and normoxic (21% O2) conditions in terms of LSEC-related gene expression. It showed that hypoxic (5% O2) led to significant upregulation of LSEC scavenger receptor genes such as *STAB2* and *FCGR2B*, while differentiation medium C showed advantage for inducing the expression of *LYVE-1, STAB2, FCGR2B* and *CD36* compared with medium A and B*.* Moreover, hypoxic conditions did not have an inhibitory effect on the expression of *CD36* and *LYVE1*. (Fig. 2D). Flow cytometry analysis confirmed that approximately 80% pure LYVE-1^+^ LSEC-like cells could be generated from KDR^+^ iEPCs under hypoxia and from differentiation medium C on day 8 (Fig. 2E).

Overall, we found that KDR^+^ EPCs may be an important precursor source of LSECs. It is possible to generate high-purity LSEC-like cells from hiPSCs as rapidly as 8 days.

### Identification of novel LSEC markers with single-cell RNA sequencing

**Figure 3:**
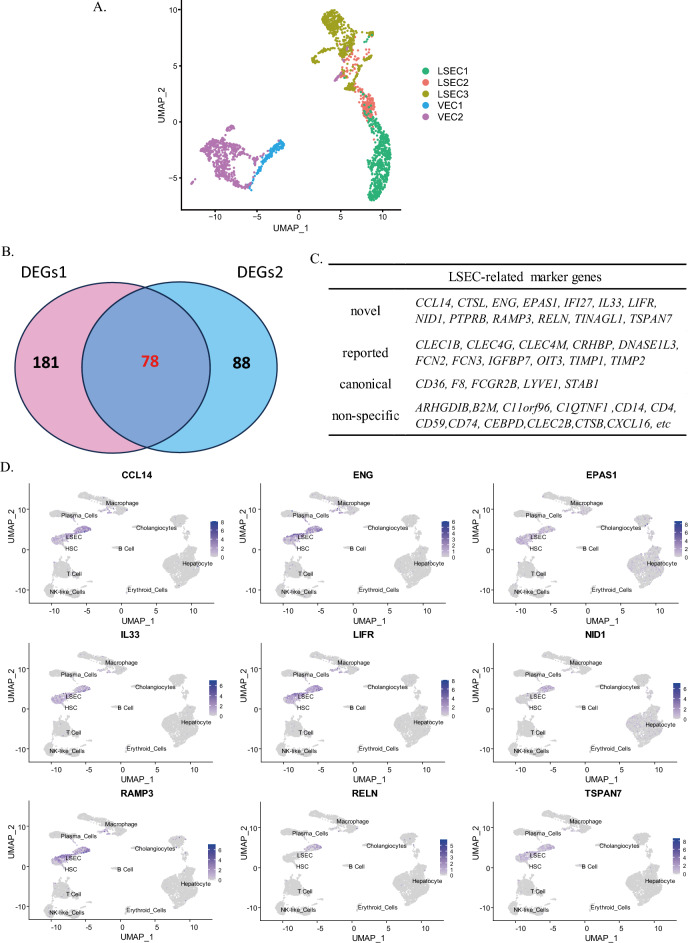
Novel LSEC-specific markers. (**A**) UMAP plot showing the relative positions of cells associated with three LSEC samples and two vascular endothelial cell (VEC) sample. (**B**) Venn diagram showing 78 intersecting genes predicted to be associated with LSECs in the two groups of DEGs. (**C**) Table showing LSECs intersecting genes. (**D**) UMAP expression plots of 9 novel LSEC-specific marker genes derived from DEG analysis of different cell types in the liver.

To further investigate LSEC-specific relevant markers, we aimed to address the current lack of LSEC markers and evaluate our differentiated LSECs. We extracted the cell populations of LSECs from three sets of published liver single-cell sequencing data, labeled LSEC1, LSEC2, and LSEC3. In addition, we examined two sets of vascular endothelial cell sequencing data, labeled VEC1 and VEC2. The sequencing data of LSECs and vascular endothelial cells were simplified to cluster integration by the UMAP algorithm. With this approach, we were able to obtain a clearer understanding of the distribution and clustering of cells in different samples (Fig. 3A). Three sets of LSEC data (LSEC1, LSEC2, LSEC3) were analyzed for differentially expressed genes separately from VEC1 data. The intersection of the results of these three comparisons was defined as different expression genes 1 (DEGs1). In the same way, we also differentially analyzed each of these three sets of LSECs data with VEC2 data, and the resulting intersection was defined as different expression genes 2 (DEGs2). There were 181DEGs1 whose expression increased more than twofold between LSECs data and VEC1 and 88 DEGs2 whose expression differed between LSECs data and VEC2 (Fig. 3B). The intersection between DEGs1 and DEGs2 was considered to indicate genes associated with the LSEC-like phenotype. There were 78 LSEC-related genes in the two groups of DEGs. We used the results of cell type identification in the liver sequencing data GSE115469 to generate a UMAP map of the distribution of liver cells populations to show specific cell distributions and to identify marker genes for our analysis. (Supplementary Fig. S1) Each of these genes was then utilized to create UMAP expression plots in the liver scRNA-seq data. The analysis revealed 49 nonspecific markers not only expressed in hLSECs but also expressed in macrophages, immune cells and HSCs, including *ARHGDIB, B2M, C11orf96, C1QTNF1, CD14, CD4, CD59, CD74, CEBPD, CLEC2B, CTSB, CTSL, CXCL16* etc.; 11 reported LSEC-related marker genes, such as*CLEC1B, CLEC4G, CLEC4M, CRHBP, DNASE1L3, FCN2, FCN3, IGFBP7, OIT3, TIMP1* and *TIMP2*^20–22^; and 5 canonical LSEC-related genes, *CD36*, *F8, FCGR2B, LYVE-1* and *STAB1*. In addition, we identified novel LSEC markers, *CCL14, CTSL, ENG, EPAS1, IFI27, IL33, LIFR, NID1, PTPRB, RAMP3, RELN, TINAGL1and TSPAN7* (Fig. 3C). The expression of all these markers was assessed by comparing the data from the human protein atlas (www.proteinatlas.org), and we found that *CTSL, IFI27, PTPRB*and *TINAGL1*were also highly expressed in other liver NPCs, not exclusively in LSECs. Finally, we screened nine novel LSEC-specific markers, which were *CCL14, ENG, EPAS1, IL33, LIFR, NID1, RAMP3, RELN and TSPAN7* (Fig. 3D).

### Characterization of iPSC-derived liver sinusoidal endothelial-like cells (iLSECs)

**Figure 4:**
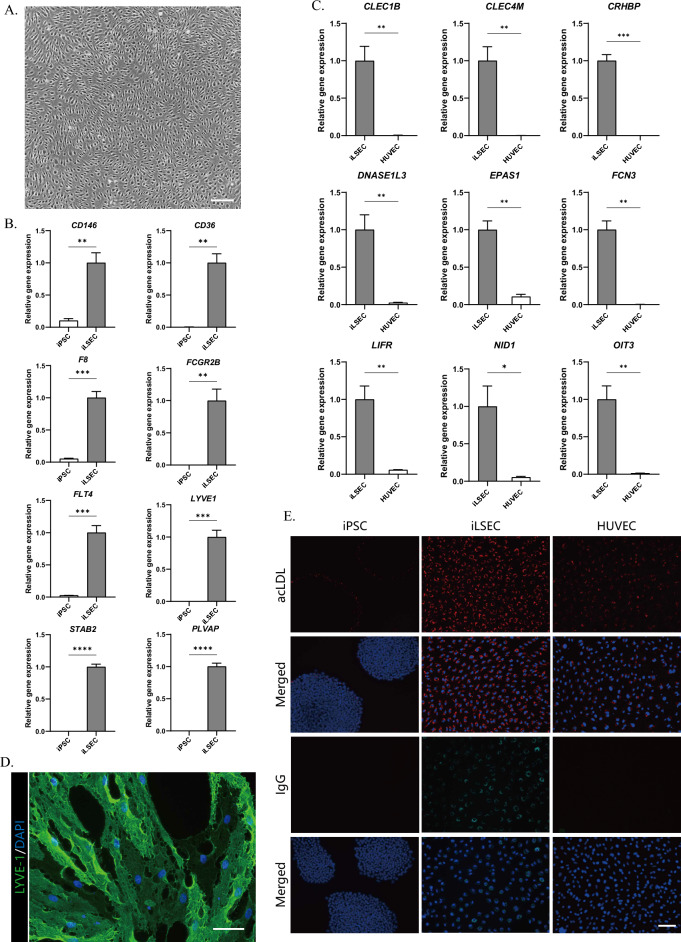
Evaluation of iLSECs. (**A**) The morphology of iLSEC-like cells after CD34 sorting. Scale bar, 100 μm. (**B**) qPCR analysis of canonical LSEC gene markers in iLSEC-like cells and HUVECs. The results are shown as the mean ± SD of 3 independent experiments. ** p < 0.01, *** p < 0.001 and **** p < 0.0001. (**C**) qRT‒PCR analysis of novel LSEC-specific marker genes (screened by ScRNA-seq analyses) in iLSECs and HUVECs. The results are shown as the mean ± SD of 3 independent experiments. * p < 0.05, **p < 0.01, ***p < 0.001 and **** p < 0.0001. (**D**) Immunostaining of LYVE-1 (green) in the iLSECs revealed irregular round or oval-shaped perforations. The nuclei were counterstained with DAPI. Scale bar, 50 μm. E Fluorescence microscopy analysis of Dil-AcLDL and CyTM5-conjugated IgG uptake. hiPSCs, purified iLSEC-like cells and HUVECs were cultured in medium supplemented with DiI-ac-LDL or CyTM5-conjugated IgG. Scale bar, 100 μm.

Following 2-step differentiation, the iLSECs exhibited a flattened and slender fusiform shape with more frequent circular surface structures, which were similar to primary human LSECs (Fig. 4A). Compared to hiPSCs, iLSECs showed significantly elevated expression of canonical LSEC genes, e.g., *CD146*, *CD36, F8, FCGR2B, FLT4, LYVE1, PLVAP* and *STAB2* (Fig. 4B). In addition, iLSECs expressed the key LSEC transcription factor c-MAF. The absence of increased expression of PDPN and PROX1 suggests that there is no induction of differentiation into lymphatic endothelial cells (Supplementary Fig. S2). We further evaluated iLSECs using the results of single cell analysis in Fig. 3. qRT‒PCR analyses revealed that the expression levels of LSEC-specific genes, such as *CLEC1B, CLEC4M, CRHBP, DNASE1L3 EPAS1, FCN3, LIFR, NID1 and OIT3,* were significantly higher in iLSECs and that these genes were hardly expressed in human umbilical vein endothelial cells (HUVECs) (Fig. 4C). Immunofluorescence imaging of LYVE-1 labeled LSEC-like cells. LYVE-1 is a lymphatic vessel endothelial hyaluronan receptor 1, has long been found to be a key LSEC marker in previous reports, and was validated by our RNA-seq analyses^23^. By immunostaining, LYVE-1 was found to be expressed in the majority of the cell populations (Fig. 4D), which is in line with our FACS results (Fig. 2E). The majority of cell populations of these LSEC-like cells presented irregular round or oval-shaped perforations with diameters of approximately 1–3 μm, which were similar to membrane fenestrations in human LSECs (Fig. 4D). LSECs are responsible for the active clearance of soluble macromolecules, small particles, immune complexes, and lipopolysaccharides^24^. In fact, LSECs are among the cells of the body with the highest endocytic capacity. The iLSECs were observed to uptake markedly more acetylated low-density lipoprotein (ac-LDL) than HUVECs and undifferentiated hiPSCs (Fig. 4E). While the capacity for endocytosis of immune complexes was observed only in the iLSECs (Fig. 4E).

Overall, these results suggested that the iLSECs had LSEC-like phenotypes and partially acquired LSEC-specific functions.

### CD34-sorting enhances the differentiation of iEPCs into LSEC-like cells

**Figure 5:**
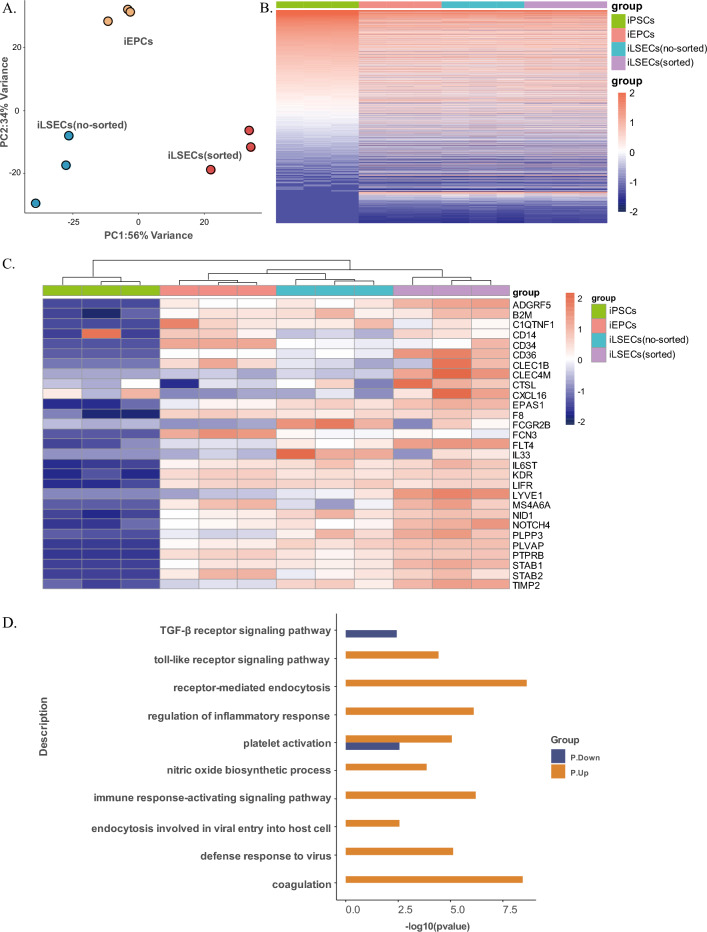
Bulk RNA sequencing of D4, D8 and enrichment of D8. (**A**) PCA analysis of iEPCs, iLSECs (no-sorted) and iLSECs (sorted) by RNA-seq. (**B**) Heatmap displaying the global gene expression profiles of iPSCs, iEPCs, iLSECs (no-sorted) and iLSECs (sorted). The color key from red to blue represents the relative gene expression level from high to low. (**C**) Heatmap of the expression profiles of LSEC-related genes in iPSCs, iEPCs, iLSECs (no-sorted) and iLSECs (sorted). The color key from red to blue represents the relative gene expression level from high to low. (**D**) The upregulation (yellow) and downregulation (blue) of genes associated with key LSEC pathways were compared between iLSECs (no-sorted) and iLSECs (sorted).

To assess the significance of CD34^+^ presorting, we compared the transcriptomes of iEPCs (Day 4) and iLSECs with or without sorting in three independent experiments. Principal component analysis (PCA) showed that iEPCs and iLSECs (no-sorted) clustered together, while there was a clear separation between iLSECs (no-sorted) and iLSECs (sorted) (Fig. 5A). This analysis indicated that iEPCs and iLSECs (sorted) exhibited remarkable transcriptome differences. We further validated the iLSECs (sorted) group by integrating the Gene Expression Omnibus (GEO) database dataset taken from the GSE119378 hiPSCs. The observed differences between iLSECs (sorted) and iLSECs (no-sorted) demonstrated that CD34 sorting on day 4 is crucial for LSEC differentiation in vitro. Different lots of induced cells clustered closely together, indicating the high reproducibility of this induction strategy. To comprehend the stepwise differentiation from iPSCs into iLSECs, the global gene expression profile revealed differences and similarities among iEPCs, iLSECs (no-sorted) and iLSECs (sorted) (Fig. 5B). To further evaluate our iLSECs (sorted), we generated a heatmap using the LSEC-related genes from Result 3 (intersecting DEGs1 and DEGs2), along with some classical genes of LSECs (Fig. 5C). The results showed that iEPCs slightly expressed LSEC-related genes, while iLSECs (sorted) displayed much wider and higher gene expression than the no-sorted cells. The pathways most enriched in the iLSECs (sorted) compared to the iLSECs (no-sorted) were examined, and those relevant to LSEC function were selected (Fig. 5D). The results demonstrated that iLSECs (sorted) showed enriched pathways involved in receptor-mediated endocytosis, coagulation, platelet activation, the Toll-like receptor signaling pathway, the nitric oxide biosynthetic process and receptor-mediated endocytosis of virus by the host cell, whereas the downregulated pathways were associated with LSEC injury, liver inflammation (TGF-β) and dysfunctional LSECs (matrix organization), which are typical features of liver fibrosis.

In summary, compared with their no-sorted counterparts, iLSECs (sorted) were involved in more pathways relevant to LSEC function, and we further confirmed that CD34^+^ iEPCs may play an important role in the successful differentiation of iLSECs.

## Discussion

In this study, we successfully developed an efficient protocol. Initially, hiPSCs were differentiated into iEPCs, and further differentiated into LSECs, termed iLSECs. This achievement not only dramatically improved the efficiency and stability of LSEC differentiation in vitro but also provided a deeper understanding and optimization of the differentiation process.

In earlier attempts, we repeatedly followed the differentiation protocol provided by Gage et al.^15^ in an effort to differentiate LSECs from an intermediate-stage hiPSC-derived endothelial cell population. However, this protocol has a long differentiation cycle and generates a large number of fibroblast-like cells during the differentiation process, which limits the generation and development of endothelial-like cells and makes it difficult to obtain LSECs stably under 2D culture conditions. Therefore, we changed and chose the earlier EPCs stage for inducing LSECs to reduce interference from other cell types. At the same time, we enriched a population of specific EPCs as precursor cells for LSEC differentiation. We demonstrated that the protocol allowed us to obtain LSECs more rapidly, efficiently, and stably in vitro.

First, in the protocol for generating iEPCs, built upon the venous endothelial cell induction protocol by Ang et al.^16^, we successfully differentiated hiPSCs into high-purity iEPCs by optimizing growth factors, especially vascular endothelial growth factor (VEGF). We found that a high concentration of VEGF is crucial for promoting the generation of EPCs, with VEGF concentration at 100 ng/ml significantly increased the proportion of CD34^+^KDR^+^ EPCs. This finding is consistent with the results showing strict dose-dependent regulation of embryonic vessel development by VEGF^25^. During the differentiation process from iEPCs to iLSECs, we referred to previous reports on the development of mouse LSECs, which are regulated by TGF-β and adrenomedullin/receptor activity-modifying protein (RAMP) signaling^13^. Combining these findings and previous reports, we selected the adrenomedullin/RAMP agonist 8-Br-cAMP, the TGF-β inhibitor SB431542, and basic fibroblast growth factor 2 (bFGF) and optimized the differentiation medium to induce iLSECs to efficiently differentiate iEPCs into LSEC-like cells that exhibited significantly increased gene expression of *CD36, FCGR2B, FLT4, LYVE1, PLVAP* and *STAB2*.

Exploring and validating the progenitor cells of LSECs during development is a key element and great challenge for optimizing differentiation protocols. In this study, we found that during the differentiation process of iLSECs, LYVE-1 was expressed mainly in the CD34^+^KDR^+^ iEPCs population, the expression of KDR gradually decreased with the differentiation process, and LYVE-1 also decreased accordingly. It is suggested that CD34^+^KDR^+^ iEPCs may be an important precursor of LSECs, which is consistent with previous reports precursor cells of LSECs expression CD34 and KDR in mice, but there is a lack of reports on human LSEC progenitors^26^. Moreover, a high proportion of CD34^+^ iEPCs play an important intermediate role in the subsequent differentiation of iLSECs. We enriched CD34^+^ iEPCs through MACS and found that the expression of *STAB2, LYVE1, FLT4* and *PLVAP* LSEC-related genes in subsequently differentiated iLSECs was increased compared with that in the untreated control. Transcriptome sequencing verified that iLSECs differentiated from CD34^+^ purified EPCs express more LSEC-related markers, with higher expression levels, and participate in a wider range of pathways related to LSEC function. The induced LSECs also displayed key functions, including ac-LDL uptake and immunoglobulin G (IgG) endocytosis, in LSECs. Notably, our optimized LSEC differentiation LSEC induction medium (LIM) significantly induced the expression of the specific LSEC markers *STAB2, LYVE1, FCGR2B* and *CD36,* especially *CD36*, which was not verified in a previous study by Gage^15^. Whereas *CD36* was found to be an important marker for LSECs, indicating that our cells have an advantage in lipid transport^27^. We also found that the hypoxic microenvironment plays a positive role in the differentiation of CD34^+^ iEPCs into LSEC-like cells by increasing the expression of LSEC-related genes, which is important for the expression of FCGR2B, a gene that is associated with the endocytosis of immune complexes, suggesting that the present protocol significantly enhances the cellular uptake of immune complexes^28^. The IgG uptake capacity was verified in this study, and gene enrichment analysis revealed that our cells were enriched in genes involved in receptor-mediated endocytosis. Furthermore, a significant advantage of our method is its simplicity, involving only a two-step induction without the need for 3D culture or other complex procedures, and time-saving differentiation, which is significantly shorter than that previously reported^14^.

Identifying specific LSEC-specific markers is crucial for a deeper understanding of the biological characteristics and functions of LSECs and their roles in liver physiology and pathology. We analyzed two sets of liver single-cell sequencing data and one set of vascular endothelial cell single-cell sequencing data from the GEO database and identified a series of known and classic genes associated with LSECs, including *CD4, CD14, LYVE1, STAB1* and *TIMP2*. The expression patterns of these genes are able to distinguish LSECs from other hepatocyte types to a certain extent but still suffer from low specificity. Notably, we also identified new potential LSEC markers, such as *CCL14, ENG, EPAS1, IL33, LIFR, NID1, RAMP3, RELN and TSPAN7*, that are capable of specifically distinguishing iLSECs from other cell types. Our differentiated iLSECs expressed most of the above-mentioned marker genes, such as *CLEC1B, CLEC4M, CRHBP* and *FCN3* which were several hundredfolds greater compared to HUVECs, while*, DNASE1L3, EPAS1, LIFR, NID1* and *OIT3*- also exhibited tens of fold greater expression. This finding suggested that our differentiated iLSECs are different from normal/regular ECs. The discovery of these markers provides a new direction for further studies on the biological functions of LSECs. However, further experimental validation and functional studies are still needed to verify the relevance of these markers in the expression pattern and function of LSECs, as well as their roles in the developmental and functional regulation of LSECs.

Nevertheless, there are still some unresolved issues, as the expression of the *F8* gene, which is functionally related to the intrinsic pathway involved in coagulation, was only marginally detected, and the *CLEC4G* and *IL33* genes, which play a role in viral and immune responses, were not significantly enhanced^29,30^. This suggests that the maturity and function of iLSECs need to be further enhanced and that a more complex environment is required to mimic LSEC development in the human liver. It has been reported that the liver-specific microenvironment, such as the extracellular matrix, liver development-related regulation of key transcription factors such as *GATA4*, signals between liver parenchyma cells and other liver cells, and interactions with the immune system, contributes significantly to LSEC development and function^31–34^. Although our study characterized iLSECs through transcriptomic analysis and in vitro functional tests, further in vivo validation and comparisons with primary human LSECs are needed to strengthen the reliability of the induction strategy. Future studies could focus on investigating the interactions between LSECs and other liver cell types, such as hepatocytes, Kupffer cells and stellate cells, to elucidate the role of LSECs in the liver microenvironment and the implications for the occurrence and progression of liver disease.

## Conclusion

In this study, we developed an efficient and reproducible two-step induction strategy to generate iLSECs from hiPSCs via the iEPC stage. A key performance should be to enrich the CD34^+^KDR^+^ subpopulation of iEPCs, which appears to facilitate the efficient induction of iLSECs. This novel induction strategy offers the potential for stable and scalable production of functional LSECs, opening new avenues for research into liver disease progression and the associated cellular microenvironment. This study also provides a basis for the screening and development of effective therapeutic drugs, exploiting the utility of hiPSC-derived iLSECs as a reliable model system.

## Materials and methods

### Culturing and maintenance of iPSCs

hiPSCs (gifts from Jiangsu University) were maintained on Matrix511-silk (Nippi 38710131) coated tissue culture plates in StemFit Basic04 medium (AJINOMOTO, Basic04CT). Cells were dissociated for passaging with Accutase (Stemcell, 07922).

### Human umbilical vein endothelial cells

Human umbilical vein endothelial cells (HUVECs, gifted from Jiangsu University) were cultured on gelatin coated plates in EGM2 (Lonza CC-3162) medium, which was refreshed every 1–2 days. Cells were used between passages 3–4.

### hiPSC differentiation into iEPCs

hiPSCs were differentiated into iEPCs in serum-free medium (SFM) as described below. The composition of SFM: 50% IMDM (GIBCO, 12440053), 50% Ham's F-12 K (GIBCO, 21127022), 1 mg/ml polyvinyl alcohol (Sigma, P8136-250G), 1% N2 (GIBCO, 17502048), 1% B27 (GIBCO, 17504044), 450 mM 1-thioglycerol (Sigma, M6145-100ML), 1% GlutaMAX (GIBCO, 35050061), and 0.1% ITS-X (GIBCO, 51500056). Day 0 hiPSCs were dissociated into single cells (Accutase) and plated into a 24-well plate in StemFit04 supplemented with Y-27632 (1 μM, Selleck) and Matrix511-silk (0.5 μl/cm^2^) at 20,000 hiPSCs/cm^2^. After 24 h, the medium was replaced with SFM supplemented with Activin A (30 ng/ml, R&D Systems), BMP4 (40 ng/ml, R&D Systems), CHIR99021 (6 μM, Tocris), or FGF2 (20 ng/ml, R&D Systems), and the cells were cultured for 24 h. Day 1 middle primitive streak cells were differentiated toward the lateral mesoderm in SFM supplemented with BMP4 (40 ng/ml), GDC-0941 (2.5 μM, Selleck), forskolin (10 μM, Tocris), SB-431542 (6 μM, Selleck), VEGF (100 ng/ml, R&D Systems), XAV939 (1 μM, Selleck) and ascorbic acid-2-phosphate (AA2P; 200 μg/ml, Sigma) for 24 h. On day 2, lateral mesoderm cells were differentiated in SFM supplemented with SB-431542 (6 μM), DMH1 (250 nM, Selleck), DAPT (10 μM, Selleck), VEGF (100 ng/ml), XAV939 (1 μM) and AA2P (200 μg/ml) for 24 h. Subsequently, day 3 cells were differentiated in SFM medium supplemented with SB431542 (6 μM), DAPT (10 μM), PD0325901 (500 nM, Selleck), CHIR99021 (1 μM) and ascorbic acid-2-phosphate (AA2P; 200 μg/ml) for 24 h, after which iEPCs were obtained on day 4.

### iEPCs differentiation into LSEC-like cells

On days 5–8, iEPCs were further differentiated into LSEC-like cells in LSEC induction medium (LIM) as described below. The composition of LIM included EGM2 (50% v/v), StemPro™-34 SFM (50% v/v, GIBCO) supplemented with 1 mg/ml polyvinyl alcohol, SB-431542 (6 μM), FGF2 (20 ng/ml), and 8-Br-cAMP (0.2 mM, Selleck). The LIM was replaced every 2 days.

Differentiation medium A consisting of 25% StemPro™-34 SFM, 75% IMDM supplemented with 0.1% ITS-X, 1% GlutaMAX, 1-thioglycerol (450 mM), ascorbic acid-2-phosphate (AA2P; 50 μg/ml), bFGF (20 ng/ml), 8-Br-cAMP (0.2 mM) and SB-431542 (6 µM); Differentiation medium B consisting of EGM-2 supplemented with VEGF (50 ng/ml) and A83-01 (1.5 µM); differentiation medium C was LSEC induction medium (LIM).

In this study, LSECs were induced under hypoxic conditions (5% CO2, 5% O2, 90% N2, 37 °C). All other cells were cultured under normoxic conditions (5% CO2, 95% air, 37 °C).

### MACS and FACS

Differentiated cells were dissociated by incubation in TrypLE Express (Gibco, 12604021) for 5 min at 37 °C. Subsequently, the dissociated cells were mixed at a ratio of 1:5–1:10 in DMEM/F12 and centrifuged (pelleted) at 300 × g for 5 min. Each cell pellet was resuspended in PBS and 2% v/v FBS (GIBCO, 10091148). EPCs were purified based on CD34 expression using mouse anti-human CD34 antibody-conjugated magnetic beads according to the manufacturer’s instructions (Miltenyi, 130046702). For FACS, the following primary antibodies were used for flow cytometric staining: anti-CD34-APC (BD Biosciences, 555824), anti-CD34-BV421 (BD Biosciences, 562577), anti-CD309-Alexa647 (BD Biosciences, 560871), anti-CD31-APC (BD Biosciences, 555445), and anti-LYVE-1-APC (R&D Systems, FAB2089). Flow cytometry was performed using a CytoFLEX (Beckman Coulter). The data were analyzed using FlowJo software (version 10.6.2).

### Quantitative PCR

The RNA isolation utilized TRIzol™ Reagent (Invitrogen, 15596018CN), and up to 1 mg of RNA was subsequently reverse transcribed into cDNA with a RevertAid First Strand cDNA Synthesis Kit (ThermoFisher, K1622). Quantitative PCR (qPCR) was conducted on a LightCycler 480 machine (Roche) using TB Green Premix Ex Taq (Takara, RR420A) along with gene-specific forward and reverse primers. The sequences of the qPCR primers used can be found in Supplementary Table 1. The expression levels of the target genes were normalized to that of the reference gene *GAPDH*.

### Immunofluorescence

The cells were fixed using a methanol and acetone mixture (1:1) at 4 °C for 30 min, followed by blocking in 10% (v/v) normal donkey serum in PBS for 1 h. Primary antibodies against CD31 (1:200, Cell Signaling, 3528S), CD34 (1:200, Abcam, ab81289), VE-cadherin (1:200, R&D Systems, AF938-SP), and LYVE1 (1:1000, Abcam, ab36993) were incubated overnight at 4 °C. Subsequently, secondary antibodies, including Cy^TM^5-conjugated donkey anti-goat (1:500, Jackson, 70575147), Cy^TM^3-conjugated donkey anti-mouse (1:500, Jackson, 715165150) and Alexa Fluor-488-conjugated donkey anti-rabbit (1:500, Invitrogen, A-21206), were applied for 60 min. Following nuclear staining with 4′,6-diamidino-2-phenylindole (DAPI) for 1–2 min at room temperature, the cells were washed three times. Fluorescently stained samples were imaged using a Revolve (ECHO) imaging system. Image processing was conducted using Image-Pro Plus (V 6.0).

### Cellular acetylated ac-LDL uptake and IgG endocytosis ability

The cells were cultured in LIM medium supplemented with 4 μg/ml Dil-acLDL (Yeasen Biotechnology, 20606ES76) for LDL uptake evaluation or 30 μg/mL Cy5-conjugated donkey anti-goat for immune complex endocytosis evaluation at 37 °C for 4 h. Then, the cells were incubated with 5 μg/ml Hoechst 33342 (Sigma, B2261) at 37 °C for 20 min after being washed with DMEM/F12, and the cells were analyzed under a Revolve (ECHO) fluorescence microscope. Images were processed for analysis using Image-Pro Plus.

### Single-cell RNA sequencing

Three liver single-cell sequencing datasets, GSE158723, GSE115469 and GSE124395; vascular endothelial cell data GSE135202 and GSE233130, were selected from the GEO database as the datasets for preliminary analysis^30,35,36^. The Seurat package was used to preprocess the data for each of these three datasets. Remove batch effects using the Harmony package. Plotting with the R package ggplot2.

### Bulk RNA sequencing

We conducted the data analysis using R software (version 4.2.2). The raw sequencing data were derived from our laboratory-generated datasets and the publicly available GEO database, encompassing the GSE 119378 dataset. Initially, data curation and preprocessing were performed using the tidyverse package (version 1.4.2.0). The DESeq2 package (version 1.4.2.0) was employed for the identification of DEGs and correction for batch effects. We established a significance threshold of p < 0.05 and a minimum fold change of 2 for the differential expression. Functional enrichment analysis of DEGs was performed using the clusterProfiler package (version 4.1.0). The enrichGO function was used for Gene Ontology (GO) enrichment analysis, and the enrichKEGG function was applied for the enrichment analysis of KEGG pathways, aiming to identify biological processes. The visualization of DEGs was facilitated by the ggplot2 package (version 3.4.4), which was used to construct bar graphs and principal component analysis (PCA) plots to intuitively display variations in gene expression. Additionally, the heatmap package (version 1.0.12) was utilized to produce heatmaps, illustrating the clustering of gene expression levels.

### Statistical analysis

Apart from bulk and scRNAseq experiments, all data are expressed as the mean ± standard deviation (SD) and were analyzed using Prism 9 (GraphPad) via one-way or two-way ANOVA with Bonferroni post hoc analysis. Significant differences were determined by Student’s two-tailed t test or Welch’s two-tailed t test depending on scedasticity. The results were considered to be significant at *p < 0.05, **p < 0.01, ***p < 0.001 and ****p < 0.0001.

### Supplementary Information

Supplementary Information.

## Data Availability

The RNA-sequencing data produced in this study is NCBI Short Read Archive: PRJNA1092415.

## References

[1] Saviano A, Henderson NC, Baumert TF (2020). Single-cell genomics and spatial transcriptomics: Discovery of novel cell states and cellular interactions in liver physiology and disease biology. J. Hepatol..

[2] Hammoutene A, Rautou PE (2019). Role of liver sinusoidal endothelial cells in non-alcoholic fatty liver disease. J. Hepatol..

[3] Poisson J (2017). Liver sinusoidal endothelial cells: Physiology and role in liver diseases. J. Hepatol..

[4] Bhandari S, Larsen AK, McCourt P, Smedsrod B, Sorensen KK (2021). The scavenger function of liver sinusoidal endothelial cells in health and disease. Front. Physiol..

[5] Tsuchida T, Friedman SL (2017). Mechanisms of hepatic stellate cell activation. Nat. Rev. Gastroenterol. Hepatol..

[6] Sakai M (2019). Liver-derived signals sequentially reprogram myeloid enhancers to initiate and maintain kupffer cell identity. Immunity.

[7] Ding BS (2014). Divergent angiocrine signals from vascular niche balance liver regeneration and fibrosis. Nature.

[8] Singhal M (2018). Endothelial cell fitness dictates the source of regenerating liver vasculature. J. Exp. Med..

[9] Ding BS (2010). Inductive angiocrine signals from sinusoidal endothelium are required for liver regeneration. Nature.

[10] Strauss O, Phillips A, Ruggiero K, Bartlett A, Dunbar PR (2017). Immunofluorescence identifies distinct subsets of endothelial cells in the human liver. Sci. Rep..

[11] Geraud C (2010). Liver sinusoidal endothelium: Amicroenvironment-dependent differentiation program in rat including the novel junctional protein liver endothelial differentiation-associated protein-1. Hepatology.

[12] Xie G (2013). Hedgehog signalling regulates liver sinusoidal endothelial cell capillarisation. Gut.

[13] Arai T (2011). Induction of LYVE-1/stabilin-2-positive liver sinusoidal endothelial-like cells from embryoid bodies by modulation of adrenomedullin-RAMP2 signaling. Peptides.

[14] Koui Y (2017). An in vitro human liver model by iPSC-derived parenchymal and non-parenchymal cells. Stem Cell Rep..

[15] Gage BK (2020). Generation of functional liver sinusoidal endothelial cells from human pluripotent stem-cell-derived venous angioblasts. Cell Stem Cell.

[16] Ang LT (2022). Generating human artery and vein cells from pluripotent stem cells highlights the arterial tropism of Nipah and Hendra viruses. Cell.

[17] Loh KM (2016). Mapping the pairwise choices leading from pluripotency to human bone, heart, and other mesoderm cell types. Cell.

[18] Loh KM (2014). Efficient endoderm induction from human pluripotent stem cells by logically directing signals controlling lineage bifurcations. Cell Stem Cell.

[19] Nonaka H, Watabe T, Saito S, Miyazono K, Miyajima A (2008). Development of stabilin2+ endothelial cells from mouse embryonic stem cells by inhibition of TGFbeta/activin signaling. Biochem. Biophys. Res. Commun..

[20] MacParland SA (2018). Single cell RNA sequencing of human liver reveals distinct intrahepatic macrophage populations. Nat. Commun..

[21] Ramachandran P (2019). Resolving the fibrotic niche of human liver cirrhosis at single-cell level. Nature.

[22] De Smedt J (2021). PU.1 drives specification of pluripotent stem cell-derived endothelial cells to LSEC-like cells. Cell Death Dis..

[23] Banerji S (1999). LYVE-1, a new homologue of the CD44 glycoprotein, is a lymph-specific receptor for hyaluronan. J. Cell Biol..

[24] Skogh T, Blomhoff R, Eskild W, Berg T (1985). Hepatic uptake of circulating IgG immune complexes. Immunology.

[25] Carmeliet P (1996). Abnormal blood vessel development and lethality in embryos lacking a single VEGF allele. Nature.

[26] Nonaka H, Tanaka M, Suzuki K, Miyajima A (2007). Development of murine hepatic sinusoidal endothelial cells characterized by the expression of hyaluronan receptors. Dev. Dyn..

[27] Su T (2021). Single-cell transcriptomics reveals zone-specific alterations of liver sinusoidal endothelial cells in cirrhosis. Cell Mol. Gastroenterol. Hepatol..

[28] Mousavi SA (2007). Receptor-mediated endocytosis of immune complexes in rat liver sinusoidal endothelial cells is mediated by FcgammaRIIb2. Hepatology.

[29] Shahani T (2014). Human liver sinusoidal endothelial cells but not hepatocytes contain factor VIII. J. Thromb. Haemost..

[30] Aizarani N (2019). A human liver cell atlas reveals heterogeneity and epithelial progenitors. Nature.

[31] Warren A (2006). T lymphocytes interact with hepatocytes through fenestrations in murine liver sinusoidal endothelial cells. Hepatology.

[32] Geraud C (2017). GATA4-dependent organ-specific endothelial differentiation controls liver development and embryonic hematopoiesis. J. Clin. Invest..

[33] Yoshida M (2007). Involvement of signaling of VEGF and TGF-beta in differentiation of sinusoidal endothelial cells during culture of fetal rat liver cells. Cell Tissue Res..

[34] DeLeve LD, Wang X, Hu L, McCuskey MK, McCuskey RS (2004). Rat liver sinusoidal endothelial cell phenotype is maintained by paracrine and autocrine regulation. Am. J. Physiol. Gastrointest. Liver Physiol..

[35] Payen VL (2021). Single-cell RNA sequencing of human liver reveals hepatic stellate cell heterogeneity. JHEP Rep..

[36] Zeng Y (2019). Tracing the first hematopoietic stem cell generation in human embryo by single-cell RNA sequencing. Cell Res..

