# Supplementary material for: A novel efficient strategy to generate liver sinusoidal endothelial cells from human pluripotent stem cells
Source: Sci Rep. 2024 Jun 15;14:13831. doi: 10.1038/s41598-024-64195-1 (PMC11180100; doi:10.1038/s41598-024-64195-1)
Supplement: Supplementary file 1 — Supplementary Information. [file 41598_2024_64195_MOESM1_ESM.pdf]

## Title Page

# A novel efficient strategy to generate liver sinusoidal endothelial cells from human pluripotent stem cells

Shang-Ping Tian[1,+], Jian-Yun Ge[1,+], Yu-Mu Song[1], Xiao-Qing Yu[1], Wen-Hao Chen[1], Yu-Ying Chen[1], Di Ye[1], and Yun-Wen Zheng[1,2,3,4,*]

**Supplementary Figure 1 The distribution of liver cell populations.**

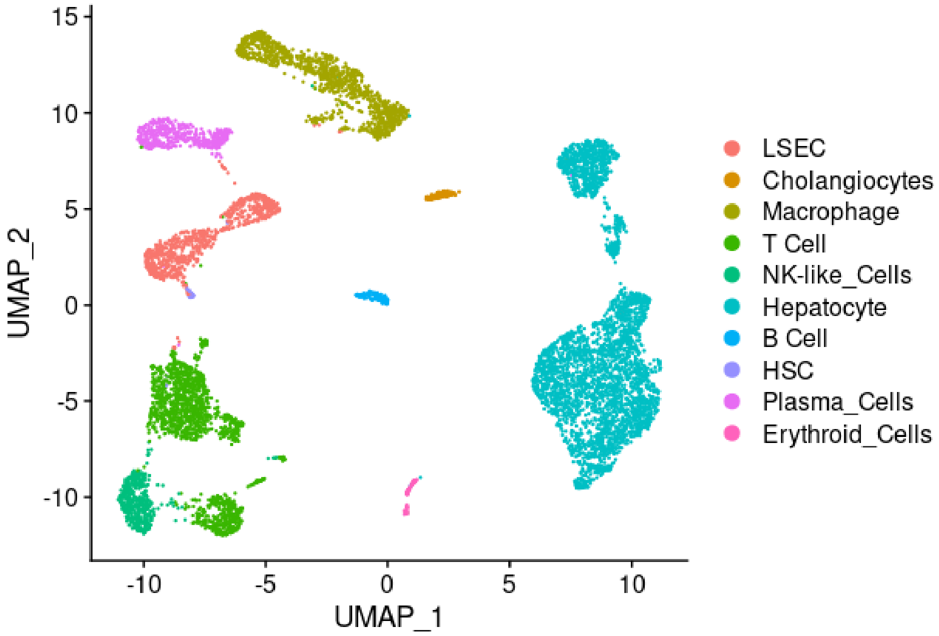


**Supplementary Figure 2***.* UMAP plot showing the relative positions liver cell populations.

**Supplementary Figure 2 The distribution of liver cell populations.**

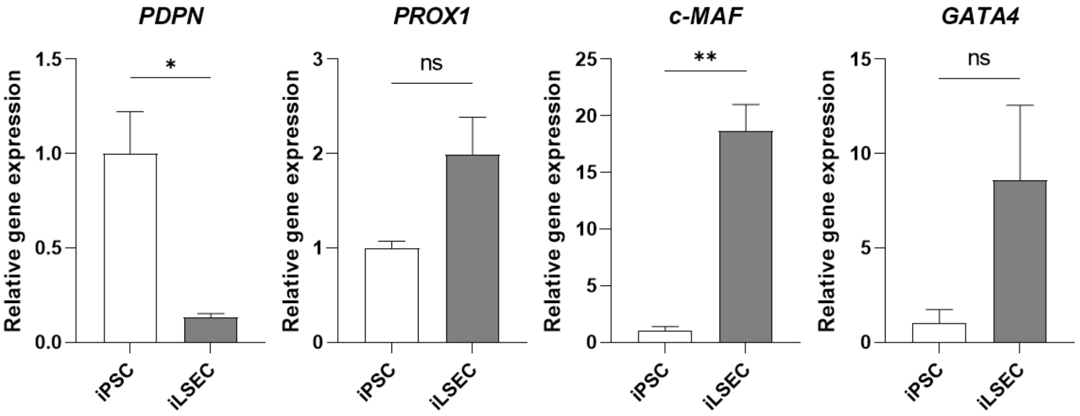


**Supplementary Figure 2.** qPCR analysis of lymphatic endothelial cella markers gene and key transcription factors for LSEC in iLSEC-like cells and iPSCs. The results are shown as the mean ± SD of 3 independent experiments. * $p < 0.05$, ** $p < 0.01$ and ns not significant.

Supplementary Table 1 RT-qPCR primer sequences

| Intended target | Gene | Forward primer | Reverse primer |
|---|---|---|---|
| Stem cells | *NANOG* | GCCGAAGAATAGCAATGGTG | AGGACTGGATGTTCTGGGTC |
| | *OCT-4* | TCTATTTGGGAAGGTATTAC | TTTCTGCTTTGCATATCTAC |
| mesodermal cells | *T* | TGCTGCAATCCCATGACAAT | CTGACTGGAGCTGGTAGGTG |
| | *SCL* | GACAGCTACACGGAGGAGCC | CAGGGTTTTCCTCGACATCTCC |
| Endothelial cells | *CDH5* | AGACTCCTTCCAGCTTCACC | GGACGCATTGAACAACCGAT |
| | *CD34* | GGCAGAAATCAAATGTTCAGGC | TCTTAAACTCCGCACAGCTG |
| | *KDR* | AATGCTCAGCAGGATGGCAA | GGAAACAGGTGAGGTAGGCA |
| | *PECAM1* | AGGAAAGAAGGACACAGAGACA | GGGAGCCTTCCGTTCTAGAGT |
| LECs | *PDPN* | AACCAGCGAAGACCGCTATAA | CGAATGCCTGTTACACTGTTGA |
| | *PROX1* | CTCGAGCCCTGATCAGAGAG | TGGCCCATGGTCTCTTTCTT |
| LSECs | *CD146* | ACCACATGAAGGAGTCCAGG | CTTCCTTCAGCATTCCCACG |
| | *CD36* | ACACTAATTCACCTCCTGAACAA | GGTCTCCAACTGGCATTAGAATA |
| | *c-MAF* | GGGTCAGCAAGGAGGAGG | GACTCCAGGACGTGTCTCTG |
| | *CLEC1B* | CTGTCCCAAGGACTGGACAT | CGTGCCTTCCTGATTTAGGT |
| | *CLEC4M* | AGACTGCATTTGAACGCCTG | CGCTGGGAGTTAGACATGAAG |
| | *CRHBP* | GGGAGGAACTGGATTGGACC | CAACTTTCATCTGGGCCGGG |
| | *DNASE1L3* | CGCTGGAAGGCGGAGAATTT | CCTTCTTGGGGACGTAGCTG |
| | *EPAS1* | CCTTGGAGGGTTTCATTGCC | AGCTCCACCTGTGTAAGTCC |
| | *F8* | CTTTTGCGATTCTGCTTTAGTGC | TAGGAGGAAATCTTGCGTCCA |
| | *FCGR2B* | CAAAGTTGGGGCTGAGAACA | CCCTGTCCTCCCCAAGGGGAA |
| | *FCN3* | CTTACTCTCCAGGGTAACTGGG | GGGCGAAAGTACGGTTACCA |
| | *FLT4* | CTCTGCCTGGGACTCCTG | GGTGTCGATGACGTGTGACT |
| | *LIFR* | GCCTCAGATATGCCCTTGGA | GCTCCAGTCACTCCACTCTT |
| | *LYVE1* | CTGCTGGGACTAAGTTTGGC | ACGAATCAATCTCCAACCCA |
| | *OIT3* | TCTTCCTGACCAACACCTCC | TTCACCACATCGACCACTGT |

| | | | |
|---|---|---|---|
| | *STAB2* | GCAAGAAGATGTGATAGGAAGTCTC | ACAACACCGAGGTTGGAGAT |
| | *GAPDH* | CAACGACCCCTTCATTGACC | CATTCTCGGCCTTGACTGTG |
